# Account of a Man Who Lives upon Large Quantities of Raw Flesh

**Published:** 1851-07

**Authors:** 


[The following history was handed to us by a friend as a medical curiosity. It was published upwards of fifty years ago in the Gazette of the United States and Philadelphia Daily Advertiser; as it may be *new* to some of our readers, we are induced to give it place.—EDS.]

*Account of a man who lives upon large quantities of raw flesh. In a letter from Dr. Johnston, Commissioner of Sick and Wounded Seamen, to Dr. Blane.*

Somerset Place, Oct. 18, 1798.

MY DEAR SIR,—Having in August and September last been engaged in a tour of public duty, for the purpose of selecting from among the prisoners of war such men as, from their infirmities, were fit objects for being released without equivalent, I heard, upon my arrival at Liverpool, an account of one of those prisoners being endowed with an appetite and digestion so far beyond anything that had ever occurred to me, either in my observation, reading, or by report, that I was desirous of ascertaining the particulars of it by ocular proof or undeniable testimony. Dr. Cochrane, Fellow of the College of Physicians of Edinburgh, and our medical agent at Liverpool, is fortunately a gentleman upon whose fidelity and accuracy I could perfectly depend, and I requested him to institute an inquiry upon this subject during my stay at that place. I enclose you an attested copy of the result of this—and as it may probably appear to you, as it does to me, a document containing facts extremely interesting, both in a natural and medical view, I will beg you to procure its insertion in some respectable periodical work.

Some farther points of inquiry respecting this extraordinary person having occurred to me since my arrival in town, I sent them in the form of queries to Dr. Cochrane, who has obligingly returned satisfactory answers. These I send along with the above mentioned attested statements, to which I beg you to subjoin such reflections as may occur to you on this subject.

I am, my dear Sir, your most obedient humble servant,

J. JOHNSTON,

To Gilbert Blane, M. D., F. R. S.,
and one of the Commissioners of Sick and Wounded Seamen.

Charles Demery, a native of Benche, on the frontiers of Poland, aged 21, was brought to the prison of Liverpool, in February, 1799, having been a soldier in the French service on board the Hoche, captured by the squadron under the command of Sir John B. Warren, off Ireland.

He is one of nine brothers, who, with their father, have been remarkable for the voraciousness of their appetites. They were all placed early in the army—and the peculiar craving for food with this young man, began at thirteen years of age.

He was allowed two rations in the army, and by his earnings or the indulgence of his comrades, procured an additional supply.

When in the camp, if bread or meat was scarce, he made up the deficiency by eating four or five pounds of grass daily—and in one year, devoured 174 cats (not their skins) dead or alive—and says he had several severe conflicts in the act of destroying them, by feeling the effects of their torments on his face and hands—sometimes he killed them before eating, but when very hungry he did not wait to perform this humane office.

Dogs and rats equally suffered from his merciless jaws—and if much pinched by famine, the entrails of animals indiscriminately become his prey. The above facts are attested by Picard, a respectable man, who was his comrade in the same regiment on board the Hoche, and is now present—and who assures me, he has often seen him feed on those animals.

When the ship on board of which he was, had surrendered after an obstinate action, finding himself, as usual, hungry, and nothing else in his way but a man's leg, which was shot off, lying before him, he attacked it greedily, and was feeding heartily, when a sailor snatched it from him, and threw it overboard.

Since he came to this prison, he has eat one dead cat and about twenty rats. But what he delights most in, is raw meat, beef or mutton, of which though plentifully supplied by eating the rations of ten men daily,* he complains he has not the same quantity, nor indulged in eating so much as he used to do, when in France.

He often devours a bullock's liver, raw, three pounds of candles and a few pounds of raw beef in one day, without tasting bread or vegetables, washing it down with water, if his allowance of beer is expended.

His subsistence at present, independent of his own rations, arises from the generosity of the prisoners, who give him a share of their allowance. Nor is his stomach confined to meat, for when in the hospital, where some of the patients refused to take their medicines, Demery had no objection to perform this for them—and his stomach never rejected anything, as he never vomits, whatever be the contents, or however large.

Wishing fairly to try how much he actually could eat in one day; on the 7th of September, 1799, at 4 o'clock in the morning, he breakfasted on four pounds of raw cow's udder—at half past 9, in presence of Dr. Johnston, commissioner of sick and wounded seaman, Admiral Child and his son, Mr. Foster agent for prisoners, and several respectable gentlemen, he exhibited his powers as follows: There was set before him five pounds of raw beef, and twelve tallow candles of a pound weight, and one bottle of porter—these he finished by half past 10 o'clock. At 1 o'clock there was again put before him, five pounds of beef, and one pound of candles, with three bottles of porter, at which time he was locked up in the room, and sentries placed at the windows to prevent his throwing away any of his provisions. At 2 o'clock, when I

* The French prisoners of war are at this time, maintained at the expense of their own nation, and are each allowed the following daily ration—twenty-six ounces of bread, half a pound of beef, half a pound of greens, two ounces of butter, or six ounces of cheese.

again saw him with two friends, he had nearly finished the whole of the candles, and great part of the beef, but had neither evacuation by vomiting, stool, or urine; his skin was cool and pulse regular, and in good spirits. At a quarter past 6, when he was to be returned to his prison, he had devoured the whole, and declared he could have eat more, but from the prisoners without telling him we wished to make some experiments on him he began to be alarmed. It is also to be observed, that the day was hot, and not having his usual exercise in the yard, it may be presumed he would otherwise have had a better appetite. On recapitulating the whole consumption of this day, it stands thus:—raw cow's udder, 4 lbs.; raw beef, 10; candles, 2. Total, 16 lbs. besides 5 bottles of porter.

The eagerness with which he attacks his beef when his stomach is not gorged, resembles the voracity of a hungry wolf, tearing off and swallowing them with canine greediness. When his throat is dry from continued exercise, he lubricates it by stripping the grease off the candle between his teeth, which he generally finishes at three mouthsful, and wrapping the wick like a ball (string and all) sends it after at a swallow. He can, when no choice is left, make shift to dine on immense quantities of raw potatoes or turnips; but from choice would never desire to taste bread or vegetables. He is in every respect healthy, his tongue clean, and his eyes lively.

After he went to the prison, he danced, smoked his pipe, and drank a bottle of porter—and, by four next morning, he awoke with his usual ravenous appetite—which he quieted by a few pounds of raw beef.

He is six feet three inches high, pale complexion, grey eyes, long brown hair, well made but thin, his countenance rather pleasant, and is good tempered.

DESTAUBAN, French Surgeon.
LE FOURNIER, Steward of the Hospital.
REVET, Commissaire de la Prison.
LE FLEM, Soldat de la ser Demi Brigade.
THOMAS COCHRANE, M. D., Inspector and Surgeon of the Prison, and Agent, &c., for sick and wounded Seamen.

*Liverpool, Sept. 9th*, 1799.

A true copy, JOHN BYNON,
Clerk in the office for sick and wounded Seamen.

*Queries and Answers.*

1. What are the circumstances of his sleep and perspiration?

He gets to bed about 8 o'clock at night, immediately after which he begins to sweat, and that so profusely as to be obliged to throw off his shirt. He feels extremely hot, and in an hour or two after he goes to sleep, which lasts until one in the morning, after which he always feels himself hungry, even though he had laid down with a full stomach. He then eats bread or beef, or whatever provision he may have reserved through the day; and if he has none, he beguiles the time in smoking tobacco. About 2 o'clock he goes to sleep again, and awakes at 5 or 6 o'clock in the morning in a violent perspiration, with great heat. This quits him on getting up; and when he has laid in a fresh cargo of raw meat (to use his own expression) he feels his body in a good state. He sweats while he is eating; and it is probable owing to this constant propensity to exhalation from the surface of the body, that his skin is commonly found to be cool.

2. What is his heat by the thermometer?

I have often tried it, and found it to be of the standard temperature of the human body. His pulse is now 84, full and regular.

3. Can this ravenous appetite be traced higher than his father?

He knows nothing of his ancestors beyond his father. When he left the country, eleven years ago, his father was alive, aged about 50, a tall, stout man, always healthy; and he can remember he was a great eater, but was too young to recollect the quantity, but that he eat his meat half boiled. He does not recollect that either himself, or his brothers, had any ailment, excepting the small pox, which ended favorably with them all. He was then an infant. His face is perfectly smooth.

4. Is his muscular strength greater or less than that of other men of his time of life?

Though his muscles are pretty firm, I do not think they are so full or plump as those of most other men. He has, however, by his own declaration, carried a load of 300 weight of flour in France, and marched 14 leagues in a day.

5. Is he dull or intelligent?

He can neither read nor write, but is very intelligent and conversable, and can give a distinct and consistent answer to any question put to him. I have put a variety at different times and in different shapes, tending to throw all the light possible on his history, and never found that he varied, so that I am inclined to believe that he adheres to the truth.

6. Under what circumstance did his voracious disposition first come on?

It came on at the age of thirteen, as has been already stated. He was then in the service of Prussia at the siege of Thionville; they were at that time much straightened for provisions, and as he found this did not suit him, he deserted into the town. He was conducted to the French General, who presented him with a large melon, which he devoured, rind and all, and then an immense quantity and variety of other species of food, to the great entertainment of that officer and his suite. From that time he has preferred raw to dressed meat; and when he eats a moderate quantity of what has been roasted or boiled, he throws it up immediately. What is stated above, therefore, respecting his never vomiting, is not to be understood literally, but imports merely that those things which are most nauseous to others, had no effect upon his stomach.

There is nothing farther to remark, but that since the attested narrative was drawn up, he has repeatedly indulged himself in the cruel repasts there described, devouring the whole animal, except the skin, bones, and bowels; but this has been put a stop to, on account of the scandal it justly excited.

In considering this case, it seems to afford some matters for reflection, which are not only objects of considerable novelty and curiosity, but interesting and important, by throwing light on the process by which the food is digested and disposed of.

Monstrosity and disease, whether in the structure of parts or in the functions of appetite, illustrate particular points of the animal economy, by exhibiting them in certain relations in which they are met with in the common course of nature. The power of the stomach in so quickly dissolving, assimilating and disposing of the aliment in ordinary cases, must strike every reflecting person with wonder, but the history of this case affords a more palpable proof, and more clear conception of these processes, just as objects of sight become sensible and striking, when viewed by a magnifying glass, or when exhibited on a larger scale.

The facts here set forth, tend also to place in a strong light, the great importance of the discharge by the skin, and to prove that it is by this outlet, more than by the bowels, that the recrementitious parts of the aliment are evacuated; that there is an admirable co-operation established between the skin and the stomach, by means of that consent of parts so observable; and so necessary, in the other functions of the animal economy; and that the purpose of aliment is not merely to administer to the growth and repair of the body, but by its bulk and peculiar stimulus to maintain the play of the organs essential to life.

